# An Ex Vivo Study of Wireless Linkage Distance between Implantable LC Resonance Sensor and External Readout Coil

**DOI:** 10.3390/s22218402

**Published:** 2022-11-01

**Authors:** Muhammad Farooq, Bilal Amin, Marcin J. Kraśny, Adnan Elahi, Muhammad Riaz ur Rehman, William Wijns, Atif Shahzad

**Affiliations:** 1 Smart Sensors Laboratory, Lambe Institute for Translational Research, College of Medicine, Nursing Health Sciences, University of Galway, H91 TK33 Galway, Ireland; 2 Electrical and Electronic Engineering, University of Galway, H91 TK33 Galway, Ireland; 3 Centre for Systems Modeling and Quantitative Biomedicine, University of Birmingham, Birmingham B15 2TT, UK

**Keywords:** physiological parameters, wireless linkage, LC sensor, bio-environment, repeater coil, implantable sensor

## Abstract

The wireless monitoring of key physiological parameters such as heart rate, respiratory rate, temperature, and pressure can aid in preventive healthcare, early diagnosis, and patient-tailored treatment. In wireless implantable sensors, the distance between the sensor and the reader device is prone to be influenced by the operating frequency, as well as by the medium between the sensor and the reader. This manuscript presents an ex vivo investigation of the wireless linkage between an implantable sensor and an external reader for medical applications. The sensor was designed and fabricated using a cost-effective and accessible fabrication process. The sensor is composed of a circular planar inductor (L) and a circular planar capacitor (C) to form an inductor–capacitor (LC) resonance tank circuit. The reader system comprises a readout coil and data acquisition instrumentation. To investigate the effect of biological medium on wireless linkage, the readout distance between the sensor and the readout coil was examined independently for porcine and ovine tissues. In the bench model, to mimic the bio-environment for the investigation, skin, muscle, and fat tissues were used. The relative magnitude of the reflection coefficient ($S_{11}$) at the readout coil was used as a metric to benchmark wireless linkage. A readable linkage signal was observed on the readout coil when the sensor was held up to 2.5 cm under layers of skin, muscle, and fat tissue. To increase the remote readout distance of the LC sensor, the effect of the repeater coil was also investigated. The experimental results showed that the magnitude of the reflection coefficient signal was increased 3–3.5 times in the presence of the repeater coil, thereby increasing the signal-to-noise ratio of the detected signal. Therefore, the repeater coil between the sensor and the readout coil allows a larger sensing range for a variety of applications in implanted or sealed fields.

## 1. Introduction

The essential physiological markers for human health monitoring include heart rate, respiration rate, temperature, blood pressure, muscle tension, and joint movement [1,2,3,4]. Human health can also be monitored by examining the electrical signal from the heart, brain, and muscles through electrocardiography (ECG), electroencephalography (EEG), and electromyography (EMG), respectively [5,6,7,8]. Currently, these signals are acquired with remarkable accuracy and reliability by adhering electrodes to the human skin with adhesive tape, needles, or mechanical clamps connected to instrumentation responsible for signal processing. These measuring instruments are also responsible for power supply, communication interfaces, and data processing, but they are tethered and bulky. This significantly reduces their portability restricting applications to bedside only. Recently, various techniques have been explored to miniaturize and increase the performance of wearable devices; however, the overall electronic systems in various solutions are still rigid, brittle, and bulky [9].

In addition to these state-of-the-art solutions, various wearable and implantable systems with miniature sensors to monitor the body parameters have been developed to record the heart rate [10], temperature [11,12,13], pH [13,14,15], pressure [16,17], blood flow [18], and respiration rates [19,20]. These solutions are flexible [21], bendable [22], stretchable [23,24,25], and biocompatible, making them suitable for both wearable and implantable applications [26,27,28].

These health monitoring sensors can be either connected wired or wirelessly to the data acquisition units [29]. The wired systems are not well suited for long-term health monitoring since they can restrict patient/user movement. In most health monitoring solutions, especially with implantable devices, wireless communication between the implanted device and the external readout system is preferred [18,22]. There have been reported very compact and biocompatible implantable antennas for wireless biomedical applications [30,31,32]. A multilayered spiral antenna covering the medical implant communication system (MICS) and industrial, scientific, and medical (ISM) bands was reported for an implantable smart healthcare monitoring system [33]. Doğancı et al. [34] and Ucar et al. [35] reported the preparation of a human skin phantom to analyze the performance of implantable antennas for the implantable communication system. Many wireless sensors are built on standard communication protocols for implantable devices such as Bluetooth, WiFi, and ZigBee [36,37]. These technologies have improved linkage and data transmission capabilities; however, they have limitations due to complex device architectures and power requirements [38].

In a variety of wearable and implantable applications, wireless sensors have been deployed. For example, the distribution of force during joint movement is measured using a system of fourteen pressure transducers in the femoral head [4]. Shoulder implants [39], femoral implants [40], and vertebral and interbody spinal implants [41] all use wireless sensors. Van Citter and Franklin reported a long-term telemetric implantable blood pressure sensor which was validated in vivo for more than a year on various mammals [42]. 

Wireless sensors can be further categorized as active or passive sensors [38,43]. The active sensors are mainly composed of a power supply unit (i.e., battery or energy harvesting transducers [44]) and electronic modules for signal processing [38]. These sensors can provide superior performance over passive sensors, but their construction is more complex, and they require a power source to work. Therefore, active sensors are not suited for long-term implanted applications due to the necessity of a power source. Some active medical devices that use batteries are neurostimulators, cochlear implants, pacemakers, cardiac defibrillators, cardiac resynchronization devices, medication delivery systems, and bone growth generators [45,46].

Passive sensors, unlike active sensors, do not require an internal power source to work because no active components are used in their design, making them more suited for long-term health monitoring [45]. Additionally, passive sensors have the advantage of adaptation and miniaturization to suit a wide range of applications. The most common examples of passive sensors include chip-less radiofrequency identification sensors (RFIDs) [47] and inductor–capacitor (LC) resonance sensors [17,43,48]. 

In recent decades, substantial research has been reported on the use of wireless LC sensors in health monitoring applications. Sridhar et al. [14] developed an LC sensor for wound healing application, based on pH level investigation. Karipott et al. [49] demonstrated the capability of LC sensors to monitor the temperature. Deng et al. [50] proposed an LC sensor to monitor the moisture level during wound curing by incorporating graphene oxide moisture-sensitive material in the sensor. Farooq et al. [51] reported an LC sensor to monitor the pressure during compression therapy. Due to the numerous sensing capabilities, wireless LC sensors have a huge potential in implantable medical applications [22,51,52,53].

The principal operation of LC-based sensors is the change in the sensors’ resonance frequency due to the induced capacitance change. For implantable medical applications, an external readout (inductive) coil is required to wirelessly power the LC sensor [54,55] and retrieve the sensors’ measuring parameters. The LC sensor resonates when exposed to an electromagnetic field. The resonance frequency in the LC system occurs when the inductive and capacitive reactance reaches its maximum value, resulting in a minimum value of the reflection coefficient [47,56]. The reflection coefficient values are measured by using the vector network analyzer (VNA). Therefore, changes in the minima in the reflection coefficient measured using a VNA reflect the change in the sensor’s resonant frequency [57]. The strength of the wireless coupling between the sensor and the readout coil is inversely proportional to the distance between them. Thus, the amplitude of the reflection coefficient decreases as the coupling strength decreases [58].

Recent studies have found that one of the key challenges for implantable LC sensors is the inductive coupling distance between the sensor and the readout coil [58,59,60]. The geometrical restrictions such as size, orientation, and non-invasiveness of the readout coil contribute to this key challenge. Studies have shown that adding a ferrite core at the center of the inductor confines the magnetic field, resulting in a stronger coupling between the sensor and the readout coil, thus increasing the readout distance [61]. This distance can be further increased by adding a repeater coil between the sensor and the readout coil. The magnetic field from the sensor is received by the repeater coil and relayed to the readout coil, increasing the coupling distance between the sensor and the readout coil [60]. 

The coupling strength between the implanted sensor and the readout coil also depends on the material properties between the sensor and the readout coil. For implantable medical devices, various tissue layers can be present between the sensor and the readout coil [45,62]. The tissue layers absorb a portion of the electromagnetic field (EM) as it propagates through the biological tissue, which is characterized by the specific absorption rate (SAR). The amount of SAR is determined by the type of tissue and the operating frequency because SAR increases rapidly with increased operating frequency [63,64].

The sensors reported in our previous research were only suitable for wearable applications, as the sensors were neither biocompatible nor waterproof and were bigger in size. Moreover, the results presented at an earlier work were for a free space environment inside a glass pressure bottle. There was no investigation presented related to wireless linkage distance in a tissue environment.

This study designed and fabricated a biocompatible LC sensor with a two-layered planar inductors approach. The proposed sensor is designed for an implantable application, reporting a small size and optimization for implantable applications, compared to the previously reported sensor for wearable applications. Due to the two-layer inductor approach, the sensor showed a lower resonance frequency, which is important for better signal penetration in the tissue environment. The wireless linkage or coupling distance between the proposed sensor and the readout coil was investigated in the presence of various tissues. Initially, the wireless linkage of the LC sensor and readout coil was investigated for fat, muscle, and skin tissue layers, and later a repeater coil was used to increase the coupling distance between the sensor and readout coil. Similar sets of experiments were performed for porcine and ovine tissues and the magnitude of the reflection coefficient at the readout coil was compared in the presence and absence of the repeater coil. 

The remainder of this paper is structured as follows: Section 2 describes the design, fabrication and validation methodologies; Section 3 describes the results and discussion while Section 4 presents the conclusion and future work.

## 2. Materials and Methods

**Figure 1:**
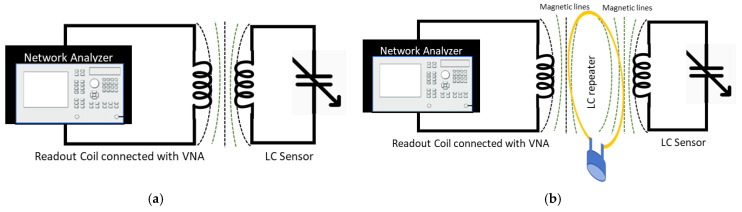
A Schematic of the LC sensing system with the readout coil connected with a VNA. (**a**) Sensing system diagram without a repeater coil. (**b**) System diagram with a repeater coil used to increase the measurement distance between the sensor and the readout coil.

The LC sensor proposed in this study is inductively coupled and wirelessly powered through the external readout coil. Figure 1a depicts a schematic of the overall wireless sensing system without the use of a repeater coil, whereas Figure 1b depicts the use of a repeater coil. The shift in the sensor’s resonance frequency is used to record the changes in the physiological parameter. The main focus of this research is to investigate the wireless linkage distance between the readout coil and sensor in the tissue environment. The proposed sensing system can be used in wearable applications such as wound monitoring and compression therapy. The reported sensor in this work is waterproof and biocompatible, therefore it can also be used for implantable applications such as intracranial pressure and intra-vascular pressure monitoring.

### 2.1. Sensor Design

**Figure 2:**
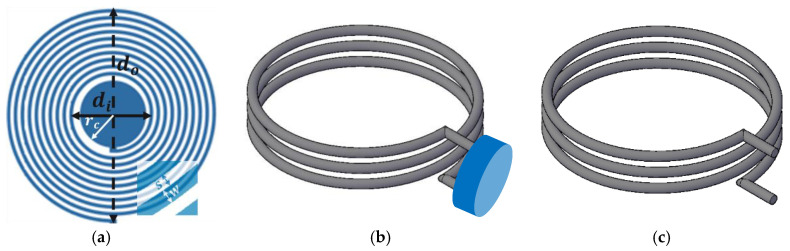
(**a**) A geometrical representation of a circular LC sensor with an outer diameter $\left(d_{o}\right)$, inner diameter $\left(d_{i}\right)$, trace width (w), trace separation (s), and electrode with radius $\left(r_{c}\right)$. (**b**) Repeater coil connected with a fixed value capacitor (shown in blue). (**c**) Reader coil with terminals opened for VNA connection.

For this study, we designed the resonance sensor consisting of a variable capacitor $\left(C_{s}\right)$ (whose value changes in a response to the applied pressure) and a fixed planar inductor $\left(L_{s}\right)$. The geometrical representation of the circular planar LC sensor is shown in Figure 2a. The inductance and capacitance combine to cause the circuit to resonate at a frequency known as the resonance frequency $\left(f_{o}\right)$, which can be calculated using Equation (1).

(1) $$f_{o}=\frac{1}{2\pi \sqrt{L_{s}C_{s}}}$$

The capacitive part is shown with the circular solid disk and the inductive part is shown with spiral traces. Equation (2) can be used to calculate the capacitance $\left(C_{s}\right)$ of the sensor:

(2) $$C_{s}=\frac{\epsilon_{o}\epsilon_{r}\;\pi r_{c}{}^{2}}{d}$$

where $r_{c}$ is the radius of the electrodes which are separated by distance d, and $\epsilon_{o}$ is free space permittivity. The permittivity of the PDMS exhibits a negligible change across the frequency range of interest in this study, as reported in [65,66,67]. Therefore, the value of PDMS relative permittivity $\epsilon_{r}\;$= 2.65 at 1 MHz has been used in this paper.

Equation (3), which is known as a current sheet expression [68], is widely used to calculate the inductance of the planar inductor. The inductance of the planar inductor depends on the inner diameter $\left(d_{i}\right)$, outer diameter $\left(d_{o}\right)$, and the number of turns (N).

(3) $$L_{s}=\frac{{µ}_{o}\cdot N^{2}\cdot d_{mean}\cdot C_{1}}{2}\left(\ln\frac{C_{2}}{\kappa }+C_{3}\cdot \kappa +C_{4}\cdot \kappa^{2}\right)$$

where $d_{mean}=\frac{(d_{i}+d_{o\;)}}{2}$, $\kappa =\frac{\left(d_{0\;}-\;d_{i}\right)}{\left(d_{o\;}+d_{i}\right)}$ and ${µ}_{o}$ is the permeability of the free space which is $4\pi \times 10^{-7}Hm^{-1}$. The values of the current sheet coefficients for the circular planar inductor are $C_{1}=1$, $C_{2}=2.46$, $C_{3}=0$ and $C_{4}=0.2$. The details of the derivation of these coefficients are provided in [68].

### 2.2. Sensor Fabrication

**Figure 3:**
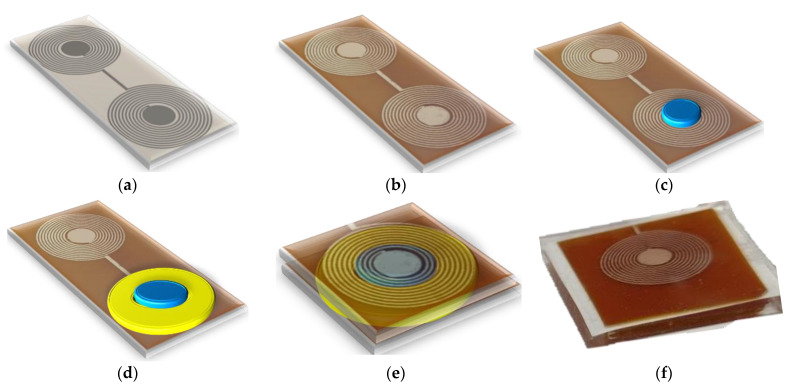
Step-by-step fabrication process: (**a**) Ink mask printed on copper-coated polyimide film. (**b**) Etched sensor patterns planar capacitor and inductor. (**c**) Circular PDMS layer as a dielectric layer on capacitor electrode. (**d**) Adhesive layer deposition. (**e**) PDMS encapsulated sensor. (**f**) Fabricated LC sensor.

The sensor was fabricated using a cost-effective and simple fabrication method. The stepwise fabrication stages are shown in Figure 3. In the first stage, as shown in Figure 3a, the mask designed in AutoCAD 2020 was printed directly on the single-sided copper-coated polyimide sheets (C.I.F. AN10 flexible isolating raw copper PCB) using a LaserJet printer (HP M553, HP Technology, Dublin, Ireland). In the next step, these mask-printed sheets were attached to a PCB holder inside a bubble etching tank (Proma 141 040 2000 Etch station) filled with sodium persulphate (Fortex Engineering Limited, Fine etch crystals 600-014) etchant (10:1 water to sodium persulfate ratio) for 15 min at 45 °C. After the completion of the etching process, sensors were removed from the tank and the residual etching solution was removed with a hot water washing process. The remaining mask ink was cleaned in the acetone bath. The etched coil pattern is shown in Figure 3b. In the next stage, a circular disk of 200 µm thick PDMS (Ultra-thin film, 30° shore A hardness, Silex Ltd., Bordon, UK) layer was cut and placed on the bottom electrode of the sensor capacitor, as shown in Figure 3c. In the next step, a 90 µm thick adhesive layer composed of synthetic rubber and polypropylene (Tesa64621, Tesa, Norderstedt, Germany) was placed around the PDMS circular disk, as shown in Figure 3d. In the next step, the top electrode was carefully folded on the PDMS layer and the bottom electrode to make the final assembly of the sensor, as shown in Figure 3e. To make the sensor biocompatible and waterproof, the sensor was encapsulated in PDMS using dip coating, as described in [69]. The image of the fully fabricated sensor is shown in Figure 3f. The key design parameters and electrical characteristics of the implanted sensor are listed in Table 1.

### 2.3. Readout Coil 

As mentioned in the design section, the sensor is an electrical LC resonant tank circuit with a variable capacitor (*Cs*) and resonate at $f_{o}$. This $f_{o}$ will change by $\Delta f_{o}\;\mathrm{Hz}$ when a slight change in physiological pressure (ΔP) will cause a change in the sensor’s capacitance ($\Delta C_{s})$ [59]. Equation (4) can be used to calculate the change in resonant frequency $\Delta f_{o}$ for small enough variations in the physiological pressure (ΔP):

(4) $$\Delta f_{o}\;≅-\frac{f_{o}}{2}\;\left(\frac{\alpha \Delta P}{(C_{s})\;}\right)\;\;\;\mathrm{for}\;\Delta C_{s}\;\ll \;C_{s}$$

where α is the proportionality constant (F/mmHg). The resonant frequency of the sensor can be detected wirelessly by measuring the return loss of the readout coil. The change in the sensor’s resonant frequency is determined by monitoring the input impedance (*Z_in_*) or return loss ($S_{11}$) of the readout coil [59]. The input impedance at the terminals of the readout coil is written as:

(5) $$Z_{in}=j\omega L_{r}+R_{r}+\frac{\eta^{2}L_{r}L_{s}\omega^{2}}{j\omega L_{s}+\frac{1}{j\omega C_{s}}+R_{s}\;},$$

where ω is the angular frequency, $L_{r}$ and $R_{r}$ are the inductance and resistance of the readout coil, respectively, $L_{s}$, $R_{s}$, and $C_{s}$ are the inductance, resistance, and capacitance of the sensor, respectively. Value η is the geometry-dependent coupling strength. The value of η ranges from 0 to 1 [59]. A value of 0 indicates no coupling, while 1 indicates maximum coupling and is expressed as:

(6) η=MLrLs

where *M* is the mutual inductance between the sensor and the readout coil. The return loss ($S_{11}$) of the readout coil as a function of input impedance and the characteristic impedance ($Z_{o}$) of the measurement system is written as:

(7) $$S_{11}={\left|\frac{Z_{in}-Z_{o}}{Z_{in}+Z_{o}}\right|}_{Z_{o}}$$

In this study, an elliptical loop coil was used as the readout coil, as shown in Figure 2c. The optimized design allowed the reader coil’s imaginary part and the LC sensor’s impedance to be matched. The proposed design of the coil has shown improved signal detection as measured by the VNA. For the readout coil, the shape of the coil was elliptical having 3 turns and the diameter of the enameled copper wire was 1 mm. The major diameter was 3 cm; however, the minor diameter was 1.5 cm which means the ratio between the major to minor axis was 2. The coil was made on a 3D printed mould and later the mould was removed and an SMA connector was connected to the terminals for connection with the VNA. To improve biocompatibility and waterproofing, the coil was encapsulated in PDMS. 

### 2.4. Repeater Coil

**Figure 4:**
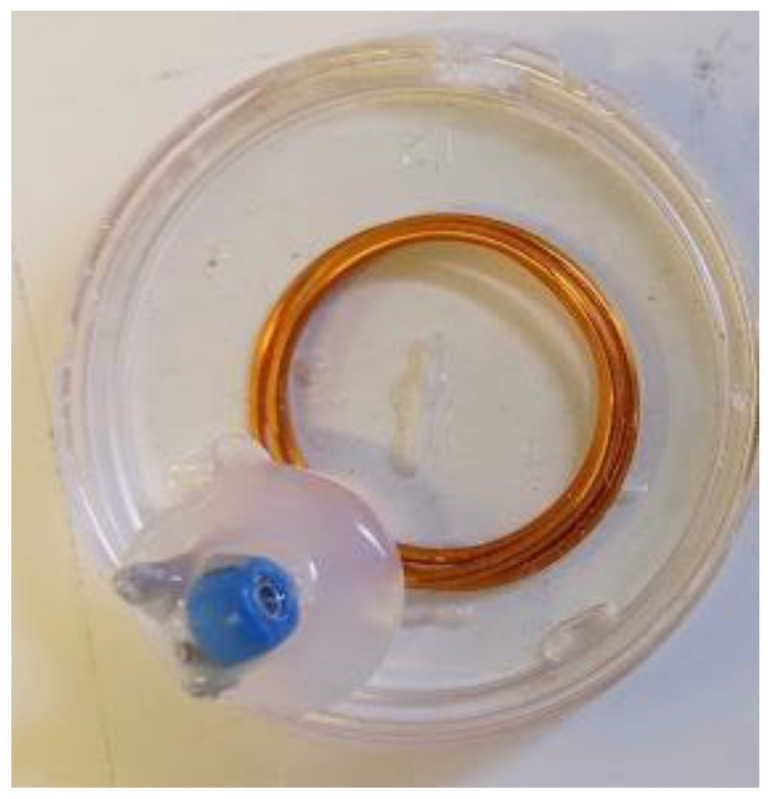
Circular repeater coil.

One of the major techniques to enhance the connectivity distance between the readout coil and the LC sensor is the use of a repeater coil [58]. The repeater coil acts as a relay between the sensor and the readout coil. The detected signal at the readout coil is maximized manyfold compared to the signal without the repeater coil [58]. The repeater and the readout coils are strongly magnetically coupled only when their resonance frequencies are matched. In this study, a circular loop coil with a parallel capacitance was used as the repeater, as shown in Figure 2b and Figure 4. 

The shape of the repeater coil was circular, having 3 turns, and the overall diameter of the repeater coil was 3 cm. The diameter of the enameled copper wire used to fabricate the repeater coil was 1.8 mm. The terminals of the repeater coil were attached to a variable trimmer capacitor ranging from 3–10 pF; however, its value was kept at 9 pF throughout the experiments. This means that the repeater coil also behaved as another LC resonant system with a resonance frequency similar to the sensor’s resonance frequency. Much like the readout coil, the repeater coil was also encapsulated in PDMS to make it biocompatible and waterproof, as shown in Figure 4. 

### 2.5. Device Validation

**Figure 5:**
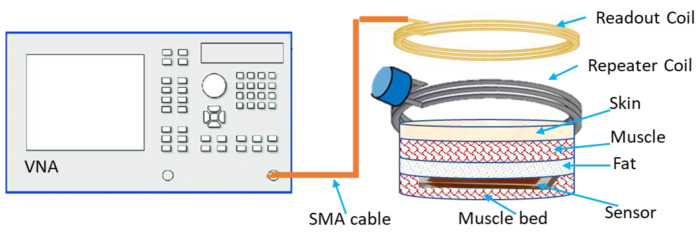
Device validation test setup.

**Figure 6:**
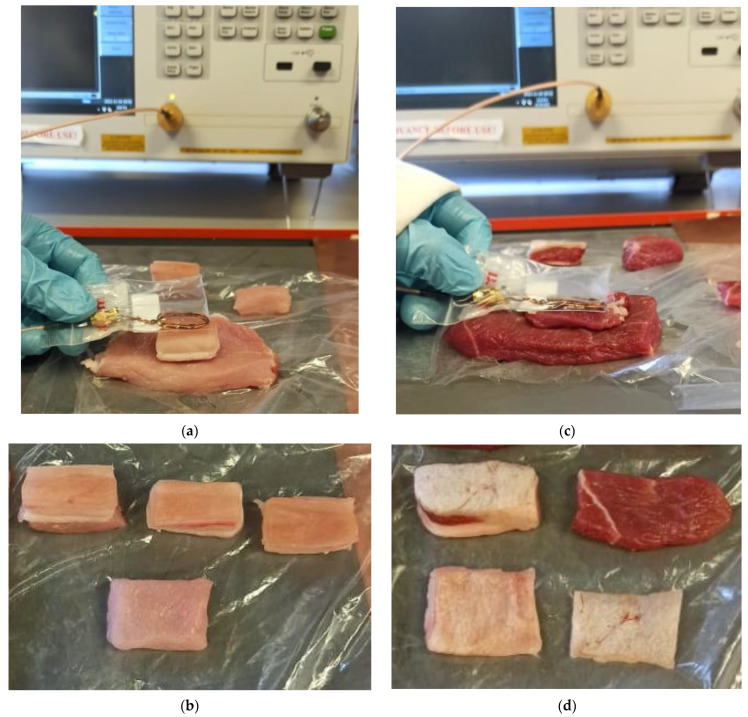
Ex vivo experiment setup for wireless linkage distance investigation. (**a**) Sensor under the porcine skin and fat layers with readout coil outside. For image purposes reader coil is shown in plastic envelope only. (**b**) Layers of different types of porcine tissue. (**c**) Sensor under the ovine muscle layer with readout coil outside. (**d**) Layers of different types of ovine tissue.

The conceptual schematic representation of the proposed system is shown in Figure 5, consisting of an implantable LC sensor, reader coil, repeater coil, SMA cable, and VNA (E5063, Keysight Technologies Inc.). The characteristic impedance of the VNA port was 50 Ω. In this study, the sensor was placed on the muscle (the biological tissues were sourced from a local abattoir) bed and covered with layers of skin, muscle, and fat and a readout coil was placed on the surface of the external tissue layer, as shown in Figure 6. This ex vivo experiment was performed to characterize the sensor and reader coil wireless linkage in the tissue environment (consisting of skin, muscle, and fat layers) prior to actual in vivo experiments. A frequency sweep of 60 to 75 MHz was generated, and the reflection coefficient $S_{11}$ was measured and recorded by using a VNA for various tissue configurations. A frequency sweep is recorded, and the location of return loss–minimum indicates the natural frequency of the sensor. Later, a similar set of measurements was recorded in the presence of a repeater coil between the sensor and readout coils.

## 3. Results and Discussion

This section separately presents the results of the characterization of the sensor and readout coil for porcine and ovine tissues. The experimental images of the ex vivo experiment are shown in Figure 6. 

### 3.1. Characterization of Sensor and Readout Coil at Porcine Tissues

To characterize the wireless linkage between the sensor and the readout coil, porcine tissues including skin, muscle, and fat were obtained from the local abattoir. The thickness of each tissue layer is listed in Table 2. An experimental setup as shown in Figure 5 was used to evaluate the readout distance between the sensor and the readout coil. 

**Figure 7:**
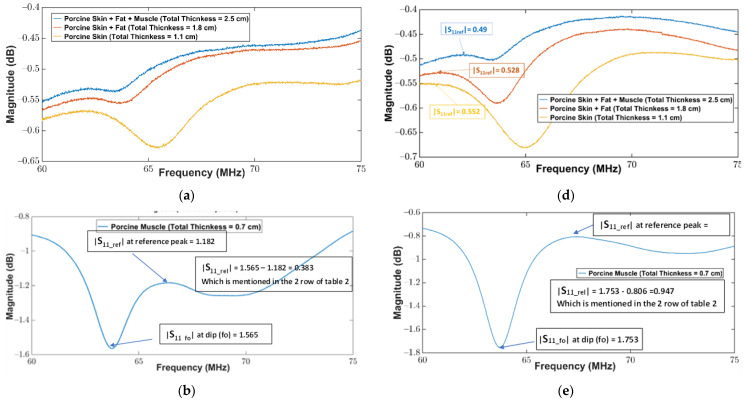

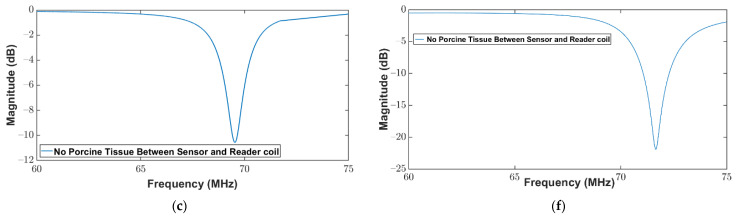
Magnitude of return loss ($S_{11}$) as a function of frequency between the sensor and the readout coil for different thicknesses of the porcine tissue layers: (**a**–**c**) without repeater coil; (**d**–**f**) with repeater coil.

For a fair comparison between the experimental results, a relative magnitude of $S_{11_{rel}}$ was computed using the absolute magnitudes of reflection coefficients at $f_{o}$ and near reference peak. The reference peak is the nearest peak from which the resonant frequency dip (minima) can be distinguished more confidently. As waveforms, Figure 7b (without repeater) and Figure 7e (with repeater) look similar; however, if the absolute magnitude at the resonance point is compared to the nearest peak (reference peak), it can clearly be seen that waveform in 7e is much better than the waveform in Figure 7b. Hence, all the analyses were made on the relative to nearest peak (reference peak) $S_{11_{rel}}$, which were calculated using Equation (8). Figure 7b,e are marked accordingly to expose the $S_{11_{rel}}$ information for additional clarification for the reader.

(8) $$\left|S_{11}{}_{rel}\left|=\right|S_{11_{fo}}\left|-\right|S_{11_{ref}}\right|$$

where ($S_{11_{fo}}$) is the value of the reflection coefficient at $f_{o}$, ($S_{11_{ref}}$) reflection coefficient at near peak, and ($S_{11_{rel}})$ relative reflection coefficient.

The resonance frequencies were measured using a VNA connected to the readout coil. In this study, the resonance frequency of the wireless sensor was approximately 69.5 MHz, as shown in Figure 7c. These experiments aimed to examine the impact of the repeater coil on the wireless linkage between the sensor and the readout coil in the presence of different thicknesses of the tissue layers. The return loss at the readout coil was measured in the presence and absence of the repeater coil for the combination of different thicknesses of the tissue layers. The measurement results are listed in Table 2. The return loss in free space was found to be maximum, as there was no tissue between the sensor and the readout coil. To validate the wireless linkage between the sensor and the readout coil, the thickness of the tissue layers was incrementally increased, as can be observed in Table 2. Initially, a muscle layer with a thickness of 0.7 cm was placed between the sensor and the readout coil. Two sets of measurements were performed for the return loss. Firstly, the return loss was measured when there was no repeater coil between the sensor and the readout coil. The second measurement was performed by adding a repeater coil between the muscle layer and the readout coil. The return loss was improved by 85% when the repeater coil was added between the muscle and the readout coil. Thus, improving the quality of the received signal in the presence of the repeater coil. Similar measurements were performed for other tissue layers as well. It can be observed from Table 2 that in the presence of the repeater coil the return loss measurements have shown significant improvement. To find the maximum linkage distance between the sensor and the readout coil all the tissue layers were combined, thus the total thickness of the tissue layers was 2.5 cm. The readout coil detected the sensor for tissue thickness of 2.5 cm. It was found that in the presence of the repeater coil the return loss was improved by 44%. Thus, the detection of the received signal was significantly improved in the presence of the repeater coil for a maximum distance of 2.5 cm between the sensor and the readout coil. 

Figure 7 shows the magnitude of the return loss against the frequency for porcine tissues. As stated above, two sets of measurements were performed for each combination of the tissue layer. When comparing the measurements in Figure 7, it can be observed that the absolute magnitude of the reflection coefficient at the resonance frequency ($S_{11_{fo}}$) is significantly higher than the absolute magnitude of the reflection coefficient at the nearest reference peak ($S_{11_{ref}}$) when repeater coils were used between the sensors and readout coil, versus without repeater coil. Therefore, the values of ($S_{11_{rel}}$) are higher with repeater coil measurements than without repeater coil measurements. Further, it can be observed from Figure 7a,d that the return loss minimum is detectable for the worst-case scenario (total thickness of all tissue layers is 2.5 cm) where all tissue layers were considered. The difference in the magnitudes of the return loss for each tissue layer was mainly observed because all the tissues in the experiments (muscle, skin, and fat) have different dielectric properties which cause a different attenuation of the applied electromagnetic field [70]. Moreover, it can be observed from Figure 7 that the resonance frequency decreased in the presence of the tissue layers compared to the resonance frequency when the sensor was placed inside the air. This is mainly because the dielectric constant of the tissue layers is much greater than the dielectric constant of the air, therefore, when a sensor is placed under the tissue layers, the parasitic capacitance between the adjacent traces becomes significantly high due to the increase of the dielectric constant in the tissue environment. This increase in parasitic capacitance decreases the quality factor of the inductive part of the sensor, as well as decreases the resonance frequency of the sensor in the tissue environment, as shown in Table 2. From the above measurements, it was observed that the proposed sensor can be detected with the proposed readout system for a maximum distance of 2.5 cm. At a depth of 2.5 cm, the variations in the capacitance were detectable in the form of a change in the resonance frequency due to the change in the parameter of interest.

### 3.2. Characterization of Sensor and Readout Coil for Ovine Tissues

To further characterize the wireless linkage and to validate the working of the sensor and the readout coil under diverse animal tissue models, ovine tissues including skin, fat, and muscle were obtained. The thickness of each tissue layer is listed in Table 3. To evaluate the change in resonance frequency with the change in the parameter of the interest on the sensor for ovine tissues, the same experimental setup was used as shown in Figure 6. The purpose of these experiments was to examine the fidelity and sensitivity of wireless linkage for tissues of different thicknesses and from different animals. The return loss at the readout coil was measured in the presence and absence of the repeater coil for the combination of different thicknesses of the tissue layers. The measurement results are listed in Table 3. It can be observed from Table 3 that in the presence of the repeater coil the return loss measurements have shown significant improvement. To find the maximum linkage distance between the sensor and the readout coil, all the tissue layers were combined, thus the total thickness of the tissue layers was 1.2 cm. The readout coil detected the sensor in the presence of different tissues (skin, fat and muscle) for a total tissue thickness of 1.2 cm. It was found that in the presence of the repeater coil the return loss was improved by 117%. The return loss measurements for ovine tissues showed consistency when compared with porcine tissues. The total thickness of all porcine tissues was 2.5 cm whereas the total thickness of tissue layers for ovine tissues was 1.2 cm. The percentage difference between the return loss for 2.5 cm thick porcine tissues and 1.2 cm thick ovine tissues in the presence of the repeater coil was found to be 166%. This shows that the thicker the tissue layers between the implanted sensor and the readout coil, the lower will be the return loss signal. Therefore, for medical implant applications, the distance between the sensor and the readout coil should be kept minimum for better detection of the return loss signal. 

**Figure 8:**
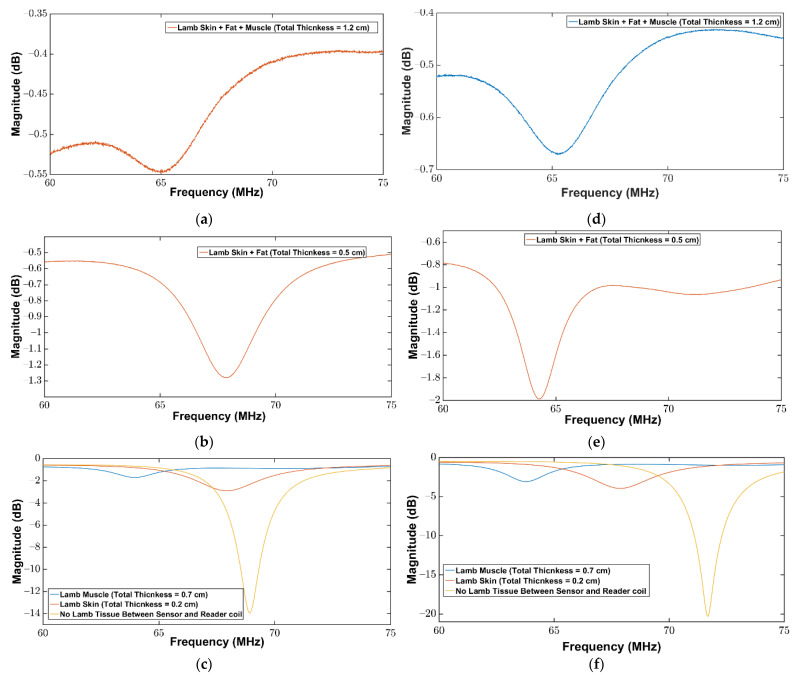
Magnitude of return loss ($S_{11}$) as a function of frequency between the sensor and the readout coil for different thicknesses of the ovine tissue layers: (**a**–**c**) without repeater coil; (**d**–**f**) with repeater coil.

Figure 8 shows the magnitude of the return loss against the frequency for ovine tissues. When comparing the measurements in Figure 8, it can be observed that the minimum in the magnitude of the return loss is noticeable for measurements where a repeater coil was used. Furthermore, it can be observed from Figure 8a,d that the return loss minimum is detectable for the worst-case scenario (total thickness of all tissue layers is 1.2 cm) where all tissue layers were present between the sensor and the readout coil. Moreover, it can be observed from Figure 8d that the return loss minimum is more prominent and well-defined when measured by incorporating the repeater coil between the sensor and the readout coil compared to Figure 8a where no repeater coil was used. When compared to measurements for porcine tissue layers, the return loss measurement results are comparable and reliable. The return loss signal is more apparent and well-defined when a repeater coil is used between the sensor and the readout coil in both porcine and ovine tissues, according to the above measurements. Furthermore, when the thickness and heterogeneity of the tissues between the readout coil and the sensor increase, the observed return loss signal weakens, making detection of the implanted sensor more challenging.

The above measurements for porcine and ovine tissues showed consistency in terms of measured return loss. The major challenge as observed in the above measurements was the presence of different tissue layers. The return loss signal, in this case, is very weak both in the presence and absence of the repeater coil. This is mainly because of the heterogeneity of tissue layers that exhibits more attenuation than the homogeneous layers [71]. Because of the heterogeneous nature of biological tissues, the electromagnetic field distribution is complicated and depends on a variety of factors, including the dielectric characteristics of each tissue and the existence of several interfaces (e.g., air/tissue and tissue/tissue) [70,72]. Moreover, the reflected and transmitted energy of the EM wave through surfaces between various tissues is determined by the relative permittivity, conductivity, and frequency [72]. The electrical properties of considered tissue (skin, fat and muscle) reported after Gabriel et al. [73] and listed in Table 4, were considered for discussion purposes.

As it can be observed from Table 4 that both the muscle and skin have higher permittivity and conductivity, therefore, the majority of the incident wave’s energy is absorbed in the skin layer and only a small portion of the energy reaches to the deeper tissue layers. The proposed sensor and the readout system, including the repeater coil, showed a maximum wireless linkage when the sensor was under 2.5 cm-thick combined tissue layer (1.1 cm (skin) + 0.7 cm (fat) + 0.7 cm (muscle)). Moreover, the proposed device has shown that fidelity and linkage between the sensor and readout coil are independent of the animal species. 

## 4. Conclusions

In this study, we designed and fabricated a biocompatible LC sensor with a two-layer planar inductors approach for implantable medical applications, using a low-cost fabrication process. The sensor was encapsulated with a PDMS layer to improve its biocompatibility. To examine the wireless coupling distance between the sensor and the readout coil, different ex vivo tests were performed on porcine and ovine tissues.

The tissue thickness between the sensor and the readout coil was varied by using different thicknesses of skin, muscle, and fat layers. The reflection coefficient was measured and recorded using an elliptical readout coil connected to the VNA. The magnitude of the reflection coefficient was found to drop sharply as the thickness of the tissue layers was increased. Moreover, the drop in the reflection coefficient was found to be different for different tissue layers, as of the differences in their dielectric properties. According to the findings of this study, the magnitude of the reflection coefficient was improved in the presence of a repeater coil between the sensor and the readout coil. As a result of this enhancement, the signal-to-noise ratio was improved, and thus the exact resonance frequency may be retrieved with greater confidence. According to the measured data, the magnitude of the reflection coefficient was improved 3.5 times and 3.75 times in the presence of combined tissue layers (skin, fat and muscle) with a maximum thickness of 2.5 cm for porcine tissues and 1.2 cm for ovine tissues. As a result, by adding a repeater coil between the implanted LC sensor and the readout coil, the confidence in obtaining critical information from received signals over longer distances is improved.

This investigation was conducted in ex vivo settings, which can aid in the optimization of the LC sensor, readout coil, and repeater coil designs before the in vivo testing. Future studies will consider different-shaped and high-quality factor readout and repeater coils. Moreover, an acyclic repeater can be used with tunable circuitry to lock the repeater coil more precisely with the LC sensor.

## Figures and Tables

**Table 1:** The key design parameters and electrical characteristics of the implanted sensor.

Parameter	Value
Number of turns, N	10
Trace width, w (mm)	0.2
Trace separation, s (mm)	0.2
$\mathrm{Outer}\;\mathrm{diameter},\;d_{o}\;\left(\mathrm{mm}\right)$	13.5
$\mathrm{Inner}\;\mathrm{diameter},\;d_{i\;}\left(\mathrm{mm}\right)$	5.5
$\mathrm{Spiral}\;\mathrm{length},\;l_{total}\;\left(\mathrm{mm}\right)$	596.9
Trace thickness, t (um)	35
Skin depth, δ (um)	7.8789
$\mathrm{DC}\;\mathrm{resistance},\;R_{fix}$ (Ω)	1.483
$\mathrm{AC}\;\mathrm{resistance},\;R_{var}\;\left(\Omega \right)$	9.0277
$\mathrm{Capacitor}\;\mathrm{electrode}\;\mathrm{radius},\;r_{c}\;\left(\mathrm{mm}\right)$	1.9
Dielectric layer thickness, d (um)	200
$\mathrm{Relative}\;\mathrm{permittivity}\;\mathrm{of}\;\mathrm{PDMS},\;\epsilon_{\mathrm{PDMS}}$	2.65
$\mathrm{Calculated}\;\mathrm{resonance}\;\mathrm{frequency},\;f_{o\_\;\mathrm{cal}}$ (MHz)	70.99
$\mathrm{Measured}\;\mathrm{resonance}\;\mathrm{frequency},\;f_{o\_\mathrm{meas}}$ (MHz)	71.5
$\mathrm{Capacitance},\;C_{s}$ (pF)	1.3305
$\mathrm{Parasitic}\;\mathrm{capacitance},\;C_{prstc}$ (pF)	1.1469
$\mathrm{Inductance},\;L_{s}$ (uH)	3.7767

**Table 2:** Magnitude of relative return loss ($S_{11_{rel}}$ ) between the sensor and the readout coil for different thicknesses of porcine tissue layers.

Tissue Layer	Thickness of Tissue Layer				Without Repeater				With Repeater			
	Skin (cm)	Fat (cm)	Muscle (cm)	Total (cm)	$f_{o}$ (MHz)	$\left|S_{11_{fo}}\right|$ (dB)	$\left|S_{11_{ref}}\right|$ (dB)	$\left|S_{11_{rel}}\right|$ (dB)	$f_{o}$ (dB)	$\left|S_{11_{fo}}\right|$ (dB)	$\left|S_{11_{ref}}\right|$ (dB)	$\left|S_{11_{rel}}\right|$ (dB)
No tissue	0	0	0	0	69.53	10.533	0.873	9.660	71.67	21.895	2.095	19.800
Muscle	0	0	0.7	0.7	63.75	1.565	1.182	0.383	63.72	1.753	0.806	0.947
Skin	1.1	0	0	1.1	65.53	0.628	0.566	0.062	64.98	0.681	0.552	0.129
Skin + Fat	1.1	0.7	0	1.8	64.01	0.556	0.545	0.011	63.69	0.590	0.528	0.062
Skin + Fat + Muscle	1.1	0.7	0.7	2.5	63.65	0.538	0.529	0.009	63.45	0.504	0.490	0.014

**Table 3:** Magnitude of relative return loss ($S_{11}{}_{rel}$ ) between the sensor and the readout coil for different thicknesses of ovine tissue layers.

Tissue Layer	Thickness of Tissue Layer				Without Repeater				With Repeater			
	Skin (cm)	Fat (cm)	Muscle (cm)	Total (cm)	$f_{o}$ (MHz)	$\left|S_{11_{fo}}\right|$ (dB)	$\left|S_{11_{ref}}\right|$ (dB)	$\left|S_{11_{rel}}\right|$ (dB)	$f_{o}$ (dB)	$\left|S_{11_{fo}}\right|$ (dB)	$\left|S_{11_{ref}}\right|$ (dB)	$\left|S_{11_{rel}}\right|$ (dB)
No tissue	0	0	0	0	68.91	13.947	0.854	13.093	71.67	20.313	1.864	18.449
Skin	0.2	0	0	0.2	67.94	2.908	0.564	2.344	67.88	3.954	0.703	3.251
Muscle	0	0	0.7	0.7	63.98	1.705	0.763	0.942	63.54	3.085	0.837	2.248
Skin + Fat	0.2	0.3	0	0.5	67.92	1.279	0.552	0.727	64.28	1.987	0.985	1.002
Skin + Fat + Muscle	0.2	0.3	0.7	1.2	65.03	0.547	0.508	0.039	65.25	0.669	0.519	0.150

**Table 4:** Dielectric properties of considered tissues. The dielectric properties are reported at 71 MHz. The values are taken from Gabriel et al. [73].

Tissue Name	Relative Permittivity	Conductivity [S/m]
Skin	86.895	0.448
Muscle	70.529	0.692
Fat	6.382	0.035

## Data Availability

Not applicable.
